# Internal traction combined with underwater endoscopic submucosal dissection for a rectal neuroendocrine tumor

**DOI:** 10.1055/a-2686-3672

**Published:** 2025-09-11

**Authors:** Juan Cao, Yang Song, De-Feng Li, Jun Yao, Ruiyue Shi, Li-Sheng Wang, Shenggang Zhan

**Affiliations:** 1 12387 Department of Gastroenterology, Shenzhen Peopleʼs Hospital (The Second Clinical Medical College, Jinan University; The First Affiliated Hospital, Southern University of Science and Technology), Shenzhen, China

Underwater endoscopic submucosal dissection (ESD) has the advantages of a clear endoscopic view and easy manipulation by buoyancy in the removal of gastrointestinal tract lesions 1 . In addition, some cases reported that traction combined with underwater ESD improved visual acuity in the removal of early pharyngeal cancer and colorectal sessile serrated adenoma/polyp 2 3 . Herein, we showed a case with a rectal neuroendocrine tumor (NET), which was successfully removed by internal traction combined with underwater ESD.

A 59-year-old female was diagnosed with a rectal NET during colorectal cancer screening examination ( Fig. 1 **a** ). Endoscopic ultrasonography (EUS) showed a hypoechoic lesion (8.3 mm × 6.6 mm) originating from the submucosa layer ( Fig. 1 **b** ). “Clip coupled with elastic ring” internal traction according to our previous study combining with underwater ESD was performed to resect the rectal NET 4 . The routine procedures involving marking the lesion, submucosal injection, circumferential incision, and clip, coupled with elastic ring internal traction, were performed ( Fig. 1 **c–f** ). Then, dissection was conducted with underwater saline immersion ( Fig. 1 **g** and Video 1 ). The lesion was successfully removed with en bloc resection, and histopathology demonstrated a neuroendocrine tumor ( Fig. 1 **h, i** ). The defect was closed with endoscopic clips ( Fig. 1 **j** ). The internal traction combined with underwater ESD may overcome the disadvantages of conventional or traction strategies in the treatment of rectal NET, such as poor visual field, deep thermal injury, and iatrogenic perforation risk. Moreover, this simple method may facilitate a constant endoscopic view and smooth continuation during the procedure; however, further studies are needed.

**Fig. 1:**
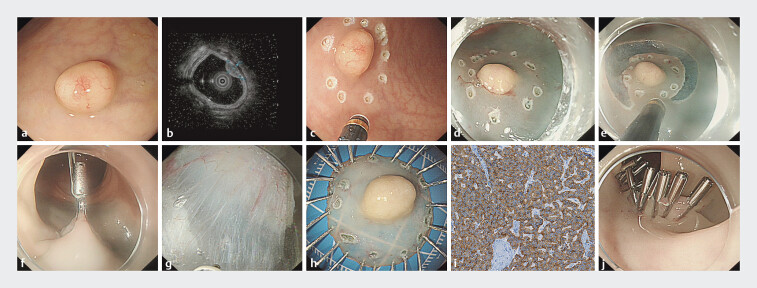
**a** A rectal NET was diagnosed during colorectal cancer screening examination. **b** EUS showed a hypoechoic lesion originating from the submucosa layer. **c** Circumferential marking was performed 5 mm outside the edge of the lesion. **d** Submucosal injection was performed around the lesion. **e** Circumferential mucosal incision was made outside the marked area. **f** Clip coupled with elastic ring internal traction was performed. **g** Dissection was conducted with underwater saline immersion. **h** The resected tumor specimen. **i** Histopathology demonstrated a neuroendocrine tumor. **j** The defect was sutured with endoscopic clips.

The procedure of internal traction combined with underwater endoscopic submucosal dissection for a rectal neuroendocrine tumor.Video 1

Endoscopy_UCTN_Code_TTT_1AQ_2AD_3AZ

## Notes

Endoscopy E-Videos https://eref.thieme.de/e-videos
*E-Videos* is an open access online section of the journal *Endoscopy* , reporting on interesting cases and new techniques in gastroenterological endoscopy. All papers include a high-quality video and are published with a Creative Commons CC-BY license. Endoscopy E-Videos qualify for HINARI discounts and waivers and eligibility is automatically checked during the submission process. We grant 100% waivers to articles whose corresponding authors are based in Group A countries and 50% waivers to those who are based in Group B countries as classified by Research4Life (see: https://www.research4life.org/access/eligibility/ ). This section has its own submission website at https://mc.manuscriptcentral.com/e-videos .

## References

[1] KongCHuangLYangMEngineering the microbiome: A novel frontier in inflammatory bowel disease treatmentChin Med J (Engl)202510.1097/CM9.000000000000356340364490

[2] Huang S Tan L Liao S Underwater endoscopic submucosal dissection with dental floss traction for the treatment of early pharyngeal cancer Endoscopy 2023 55 E1184 E1185 37984391 10.1055/a-2197-9514 PMC10659838

[3] Pinard F Jacques J Grainville T Multipolar traction pulley method combined with underwater endoscopic submucosal dissection for a large rectal laterally spreading tumor Endoscopy 2024 56 E96 E97 38290711 10.1055/a-2239-8558 PMC10827519

[4] Li H Shi RY Yao J “Clip coupled with an elastic ring” internal traction for endoscopic submucosal dissection of a rectal neuroendocrine tumor: a junior endoscopist experience Rev Esp Enferm Dig 2024 116 164 165 37073703 10.17235/reed.2023.9617/2023

